# Chromatin remodeling by the CHD7 protein is impaired by mutations that cause human developmental disorders

**DOI:** 10.1186/1756-8935-6-S1-P107

**Published:** 2013-04-08

**Authors:** Karim Bouazoune, Robert E Kingston

**Affiliations:** 1 Department of Molecular Biology, Massachusetts General Hospital & Department of Genetics, Harvard Medical School, Boston, MA, USA

## Background

Mutations in the *CHD7* gene cause CHARGE, a developmental syndrome which affect most organs. In addition, CHD7 mutations also cause puberty and reproductive organ formation disorders such as Idiopathic Hypogonadotropic Hypogonadism and Kallmann Syndrome. Genetic studies in model organisms have further established CHD7 as a central regulator of vertebrate development. To understand how the CHD7 proteins achieve its function and how mutation of CHD7 leads to developmental disorders, it is critical to characterize WT and mutant CHD7 proteins biochemically. However to date, CHD7 has not been characterized for activity, as it is extremely large and has resisted purification.

## Materials and methods

We used the baculovirus system and a dual-tag strategy to purify intact recombinant WT and mutant CHD7 proteins. We subjected these polypeptides to nucleosome remodeling and ATPase assays to characterize the CHD7 basic properties, perform a structure-function analysis of CHD7 and examine point mutants reported in human patients.

## Results

We show that CHD7 is an ATP-dependent nucleosome remodeling factor and that it has characteristics distinct from SWI/ SNF- and ISWI-type remodelers. Further investigations show that CHD7 patient mutations have consequences that range from subtle to complete inactivation of remodeling activity, raising the possibility that even partial impairment of remodeling function has a significant impact on human biology. In addition, we find that patient mutations leading to protein truncations upstream of amino acid 1899 of CHD7 are likely to cause a hypomorphic phenotype for remodeling.

## Conclusions

We propose that nucleosome remodeling is a key function for CHD7 during developmental processes and provide a molecular basis for predicting the impact of disease mutations on that function.

